# Is excess weight a burden for older adults who suffer chronic pain?

**DOI:** 10.1186/s12877-018-0963-4

**Published:** 2018-11-08

**Authors:** Huan-Ji Dong, Britt Larsson, Lars-Åke Levin, Lars Bernfort, Björn Gerdle

**Affiliations:** 1 Pain and Rehabilitation Medicine, Department of Medicine and Health Sciences (IMH), Faculty of Health Sciences, Linköping University, SE-581 85 Linköping, Sweden; 2 Division of Health Care Analysis, Department of Medical and Health Sciences, Linköping University, SE-581 85 Linköping, Sweden

**Keywords:** Chronic pain, Older adults, Obesity, Overweight

## Abstract

**Background:**

Obesity and chronic pain are common comorbidities and adversely influence each other. Advanced age is associated with more comorbidities and multi-morbidities. In this study, we investigated the burden of overweight/obesity and its comorbidities and their associations with chronic pain in a random population sample of Swedish older adults.

**Methods:**

The cross-sectional analysis involved a random sample of a population ≥ 65 years in south-eastern Sweden (*N* = 6243). Data were collected from a postal questionnaire that addressed pain aspects, body mass index (BMI), and health experiences. Chronic pain was defined as pain during the previous three months. According to the 0–10 Numeric Rating Scale, pain scored ≥7 corresponds to severe pain. Binary logistic regression was used to determine the variables associated to pain aspects.

**Results:**

A total of 2633 (42%) reported chronic pain. More obese older adults (BMI ≥30 kg/m^2^) experienced chronic pain (58%) than those who were low-normal weight (BMI < 25 kg/m^2^, 39%) or overweight (25 ≤ BMI < 30 kg/m^2^, 41%). Obese elderly more frequently had pain in extremities and lower back than their peers. In the multivariate model, obesity (Odds Ratio (OR) 1.59, 95% Confidence Interval (CI) 1.33–1.91) but not overweight (OR 1.08, 95% CI 0.95–1.22) was associated with chronic pain. Obesity (OR 1.53, 95% CI 1.16–2.01) was also significantly related to severe pain. We also found other comorbidities – i.e., traumatic history (OR 2.52, 95% CI 1.99–3.19), rheumatic diseases (OR 5.21, 95% CI 4.54–5.97), age ≥ 85 years (OR 1.66, 95% CI 1.22–2.25), and depression or anxiety diagnosis (OR 1.83, 95% CI 1.32–2.53) – showed stronger associations with pain aspects than weight status. Conclusion: In older adults, excess weight (BMI 30 or above) is a potentially modifiable factor but not the only risk factor that is associated with chronic pain and severe pain. Future studies should investigate the effectiveness of interventions that treat comorbid pain and obesity in older adults.

## Background

Chronic pain is common in older adults, but the prevalence varies widely, ranging from 20 to 93% [1]. This wide variation is likely due to the representative population samples used and discrepancies between questionnaires that assess pain. In Sweden, more than 50% of people aged 65 and over report chronic pain [2] and in the future this percentage is likely to increase in the oldest age groups [3].

The prevalence of overweight and obesity in the elderly population is increasing worldwide [4, 5]. Excess weight can affect longevity and disease-specific mortality in old age [5–7]. Rather than just surviving to an old age, more attention is now being paid to healthy aging. Both chronic pain and obesity can be barriers for healthy aging as these factors may affect important domains of life quality such as physical independence, mental well-being, and health [8, 9].

Obesity and chronic pain often occur simultaneously. The two conditions adversely influence each other [10, 11]. Both in the general population and in old age groups, increased Body Mass Index (BMI) is positively related to chronic pain [12, 13], specifically in the lower limbs (i.e., hip, leg, knee, and foot) [12, 14, 15], spine (neck and back) [14, 16], and head as manifested as headaches [13, 17]. Pain can be a barrier to weight reduction. Patients with severe pain lost less weight than those with none-to-moderate pain during a weight management program, suggesting severe pain impeded their weight loss [18, 19]. Unfortunately, current clinical practice is more likely to treat pain and excess weight as separate issues [11, 20]. The complexity of managing each condition independently means that some other factors need to be considered.

Obesity and chronic pain share some co-morbidities (e.g., osteoarthritis, hypertension, depression, and anxiety) [13, 21–23] and poor health-related quality of life [4] as well as being associated with socio-demographic factors such as being female [13], low education [24], and low socio-economic status [25]. Smoking behaviour and alcohol consumption have also been considered as determinants of the relationship between obesity and chronic pain, but the literature is not entirely in agreement [24, 26–29]. However, few studies have examined weight in relation to socio-demographic profiles, comorbidities, and lifestyle habits. Moreover, the increase in life expectancy has resulted in more age-related diseases. The combination of various chronic conditions and diseases were observed frequently in aging populations [30, 31]. A majority of older adults have comorbidities and multi-morbidities [32, 33]. A knowledge gap exists as to whether, and to which extent, multiple comorbidities might substantially contribute to the weight-chronic pain relationship in old age. Using a random population sample of Swedish older adults, this cross-sectional study investigates the weight-chronic pain association with respect to sociodemographic factors, comorbidities, and lifestyle habits.

## Methods

### Participants and procedure

Using a cross-sectional postal questionnaire, this study collected data from a stratified random sample of 10,000 older adults (≥ 65 years old) based on five age strata (65 to 69 years, 70 to 74 years, 75 to 79 years, 80 to 84 years, and 85 years and older) from the Swedish Total Population Register for the two largest cities (Linköping and Norrköping) of a county (Östergötland) in south-eastern Sweden. The questionnaire was mailed in October 2012 and, if needed, two reminders at two-week intervals were mailed. The collection of questionnaires closed in January 2013.

### Measurements

The survey included several validated instruments/scales. An overview of all parts of the survey has been presented elsewhere [34, 35]. The relevant instruments for this study are described below.

### Demographic aspects

Age, sex, educational level, and civil status were recorded from the respondents’ answers in the postal survey. Civil status was categorized as single, married, divorced, and widowed. Educational level was classified as high school (elementary/secondary), upper school, or vocational training for more than two years, college or university for one to two years, and college or university for three years or more.

### Anthropometric variables

Height and weight were recorded from the respondents’ answers in the postal survey and the BMI (kg/m^2^) was calculated using these data. Specifically, BMI was calculated as weight (kg)/height (m)^2^ and classified according to the criteria developed by the World Health Organization (WHO): < 18.5 = underweight; 18.5–24.9 = normal range; 25.0–29.9 = overweight; and ≥ 30.0 = obesity. Morbid obesity (severely obese) is defined as a BMI category above 35.0.

Several studies have compared the validity of measured weight- and height-calculated BMI and self-reported weight- and height-calculated BMI. High correlations were reported between the two measures (Pearson’s *r* = 0.89 to 0.97 for different age groups and gender) [36]. Compared to the measured BMI, self-reported BMI had a sensitivity of 88.1% and specificity of 97.4% for identifying overweight/obesity [37].

### Characteristics of pain

Pain intensity over the preceding seven days was assessed using an 11-point numeric rating scale (NRS), ranging from 0 (no pain) to 10 (worst imaginable pain) [38]. The cut-offs of NRS for definitions of mild, moderate, and severe pain vary in the literature [39–42]. In this study, NRS scored 1–3, 4–6, and 7–10 corresponded to mild, moderate, and severe pain, respectively. We selected the cut-offs based on the knowledge that moderate and severe pain make it difficult with an individual’s activities of daily living [42, 43]. The duration of pain was registered using one question with three alternatives: no; yes, with less duration than three months; yes, with a duration of more than three months. The present study reports the proportion with chronic pain – i.e., pain with a duration of more than three months.

All the respondents marked their painful site for the previous seven days on a body manikin divided into a total of 45 sections on the front and on the back [44, 45]. From these sections, we identified 23 anatomical pain sites and developed a total index to denote the number of pain sites (NPS), ranging from 0 to 23 [44]. High values indicated higher spreading of pain (multi-site pain).

### Co-morbidities

The evaluation of co-morbidities was based on a 12-item self-reported list covering different aspects of common co-morbidities: (1) traumatic accident, (2) rheumatic arthritis and osteoarthritis, (3) cardiovascular diseases (including high blood pressure, angina pectoris, and heart attacks), (4) diseases of airways or lungs, (5) low mood and depression, (6) anxiety, (7) diseases of the gastrointestinal system, (8) diseases of the nervous system, including eyes and ears, (9) diseases of the urogenital organs, (10) diseases of the skin, (11) tumours and cancer, and (12) metabolic diseases such as diabetes, obesity, anorexia, bulimia, and goitre. These co-morbidities were reported on a five-point scale: 1 = no; 2 = yes, according to both my own and my doctor’s opinions; 3 = yes, according to my own opinion; 4 = yes, according to my doctor’s opinion; and 5 = do not know. We combined the answers for 2 and 4 to increase the robustness of measurements of the presence of a certain comorbidity. Hence, these items were dichotomized as follows: yes, according to both my own and my doctor’s opinions plus according to my doctor’s opinion and the three other alternatives.

### Life style factors

From the instrument “Health Curve” (*Hälsokurvan*) [46], we chose four questions concerning smoking and snuff use that addressed both frequency (from never to daily) and number of cigarettes per day (1 to 9; 10 to 19; and 20 or more) and number of snuff boxes per week (1 to 3 per week and 7 or more per week). Five questions concerned alcohol habits. For those who confirmed alcohol consumption, the four CAGE questions (Cut-down, Annoy, Guilty, and Eye-opener) were used to screen possible alcohol addiction problems [47]; a score of ≥ 2 was considered to indicate potential problems with alcohol abuse.

### Statistical analysis

Statistical analysis was performed using the SPSS statistical package (version 22.0 IBM Inc., New York, USA). Data were reported as the mean with standard deviation (SD), the median with interquartile range (IQR), or the number with percentage based on the data distributions. Missing data were excluded from the analysis and calculated percentages were obtained from the number of valid responses. Differences among groups were assessed using the Chi-square, the one-way ANOVA, and the Kruskal-Wallis tests as appropriate. A *p*-value < 0.05 was considered statistically significant. Binary logistic regression was used to test predictors for chronic pain, moderate pain (NRS 4–6), and severe chronic pain (NRS 7–10). BMI was entered as a categorical predictor using low-normal weight as the reference group. In the univariate analysis, we examined the effect of each variable on chronic pain, including demographic aspects (age and sex), social economic factors (marital status, education, and yearly income levels), lifestyle habits (smoking and alcohol consumption), and obesity or pain-related comorbidities. In the multivariate logistic regression, a forward (likely ratio, LR) method was used by entering each variable forwardly and removing the least significant variables from the model until all remaining variables were significant (*p* < 0.05 or *p* ≥ 0.1 for entry or removal, respectively). Goodness of fit was performed using the Hosmer and Lemeshow test where a *p*-value greater than 0.05 indicated good fit of the model. Collinearity was tested using a correlation matrix of estimates. Because diagnosis of depression and anxiety showed a high collinearity (*r* = 0.608), we transformed the two variables to a new variable (1 = depression and/or anxiety) and re-tested the correlations. This new variable represents aspects of mental health.

## Results

A total of 6243 individuals completed the questionnaire including height and weight variables used to calculate BMI. For the study population, the average BMI was 25.79 ± 4.18 kg/m^2^ and 39% (2434) were categorized as overweight and 14% (871) were categorized as obese. In the obesity group, 17.3% (151) were categorized as morbidly obese. Compared with the low-normal weight and the overweight groups, the obesity group was relatively younger (< 75 years old), had a higher proportion of males, lower education, lower yearly income, more smokers, and more suspected high alcohol consumers (Table 1).

**Table 1:** Demographic characteristics, pain aspects, and prevalence of comorbidities in the three groups of weight status

	Low-normal weight *N* = 2938	Overweight *N* = 2448	Obesity *N* = 871	*P*-value (*F* or x^2^)
Age, mean ± SD	77.1 ± 7.8	75.6 ± 7.2	74.2 ± 6.5	< 0.001 (*F* = 59.5)
65–74, *n* (%)	1218 (41.5)	1160 (47.7)	479 (55)	< 0.001 (x^2^ = 111.5)
75–84, *n* (%)	1164 (39.6)	982 (40.3)	328 (37.7)	
85+, *n* (%)	556 (18.9)	292 (12)	64 (7.3)	
Female, *n* (%)	1675 (57)	1179 (48.2)	481 (55.2)	< 0.001 (x^2^ = 43.5)
Married, *n* (%)	1652 (56.2)	1442 (58.9)	497 (57.1)	0.129 (x^2^ = 4.10)
Education, *n* (%)^1^				< 0.001 (x^2^ = 31.0)
9-year Compulsory school	1393 (48.9)	1275 (53.8)	477 (57)	
Upper secondary school	749 (26.3)	614 (25.9)	211 (25.2)	
College/University	705 (24.8)	479 (20.2)	149 (17.8)	
Income (SEK per year), *n* (%)				< 0.001 (x^2^ = 21.4)
< 150,000	872 (29.7)	662 (27)	296 (34)	
150,001~ 220,000	982 (33.4)	861 (35)	307 (35.2)	
> 220,000	1084 (36.9)	925 (37.8)	268 (30.8)	
Smoking, *n* (%)^2^				< 0.001 (x^2^ = 79.7)
Never smoker	1620 (57.2)	1244 (53.1)	396 (48.2)	
Ex-smoker	917 (32.4)	955 (40.8)	368 (44.8)	
Current smoker	295 (10.4)	143 (6.1)	57 (6.9)	
High alcohol consumption, *n* (%)	112 (3.8)	118 (4.8)	55 (6.3)	0.006 (x^2^ = 10.3)
Pain aspects, *n* (%)				
Chronic pain	1134 (38.6)	1023 (41.8)	482 (55.3)	< 0.001 (x^2^ = 77.5)
NPS, median (IQR)^a,3^	3 (1–5)	3 (2–5)	4 (2–6)	< 0.001 (x^2^ = 36.1)
No pain (NRS 0)^a^	17 (1.6)	12 (1.2)	8 (1.8)	< 0.001 (x^2^ = 37.1)
Mild pain (NRS 1–3)^a^	300 (26.5)	224 (21.9)	79 (16.4)	
Moderate pain (NRS 4–6)^a^	521 (45.9)	501 (49)	217 (45)	
Severe pain (NRS 7–10)^a^	218 (20.6)	226 (23.5)	148 (32.7)	
Comorbidities, *n* (%)				
History of trauma or injury accident	190 (6.5)	183 (7.5)	64 (7.3)	0.340 (x^2^ = 2.30)
Rheumatic diseases	638 (21.7)	637 (26)	327 (37.5)	< 0.001 (x^2^ = 88.7)
Cardiovascular diseases	1242 (42.3)	1301 (53.1)	553 (63.5)	< 0.001 (x^2^ = 142.6)
Respiratory diseases	317 (10.8)	264 (10.8)	155 (17.8)	< 0.001 (x^2^ = 35.5)
Gastrointestinal diseases	411 (14)	378 (15.4)	167 (19.2)	0.001 (x^2^ = 14.0)
Neurological diseases	914 (31.1)	731 (29.9)	228 (26.2)	0.02 (x^2^ = 7.80)
Urogenital diseases	240 (8.2)	213 (8.7)	87 (10)	0.238 (x^2^ = 2.85)
Metabolic diseases	290 (9.9)	379 (15.6)	274 (31.5)	< 0.001 (x^2^ = 244.8)
Cancer diagnosis	230 (7.8)	178 (7.3)	64 (7.3)	0.723 (x^2^ = 0.65)
Depression	132 (4.5)	105 (4.3)	48 (5.5)	0.337 (x^2^ = 2.18)
Anxiety	129 (4.4)	97 (4)	47 (5.4)	0.217 (x^2^ = 3.06)

The far right column shows the result of the omnibus statistical testing (*p*-value, *F* and x^2^)

*NPS* number of pain sites; ^a^Individuals with chronic pain *N* = 2639

Missing data: ^1^ = 205; ^2^ = 262; ^3^ = 168

**Fig. 1:**
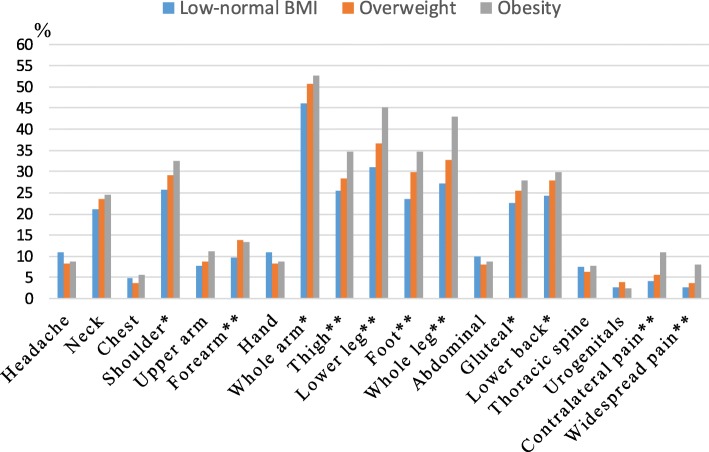
Prevalence of pain in 23 anatomical regions with BMI stratifications. The regions of head, shoulder, upper arm, forearm, hand, thigh, lower leg, and foot included both right and left sides. ^*^P < 0.05, ^**^P < 0.01

As reported elsewhere, chronic pain was reported by 51.3% of the investigated cohort [44]. More than 55% of the obese group had chronic pain, whereas 38.3% of the low-normal weight group and 41.8% of the overweight group had chronic pain (*p* < 0.001). Among the individuals with chronic pain, 32.7% of the obese group, 23.5% of the overweight and 20.6 of the low-normal weight (reported severe pain (*p* < 0.001). Similarly, according to NPS, the spread of chronic pain was more evident in the obese group (median = 4) than in the two other groups (median 3 for both groups) (*p* < 0.001). Pain in extremities and lower back were also more prevalent in the obese group (Fig. 1). A variety of co-morbidities (e.g., rheumatic diseases, cardiovascular diseases, respiratory diseases, gastrointestinal diseases, and metabolic diseases) were more frequently reported by obese individuals than their counterparts (Table 1).

Univariate logistic regression showed that higher BMI, being female, low education (nine-year compulsory school), smoking history, high alcohol consumption, and several comorbidities were positively associated with chronic pain (Table 2). Most of these positive effects remained in the multivariate regression, except for being overweight, education, yearly income, high alcohol consumption, and having cardiovascular diseases or cancer (Table 2). Being obese (OR 1.59, 95% CI 1.32–1.91, *p* < 0.001) but not overweight (OR 1.08, 95% CI 0.95–1.21, *p* = 0.254) was more likely associated with chronic pain than low-normal weight. Some factors, such as having trauma or injury accident history (OR 2.52, 95% CI: 1.99–3.19) and rheumatic diseases (OR 5.21, 95% CI: 4.54–5.97), showed higher associations with chronic pain than obesity status.

**Table 2:** Univariate and multivariate logistic regression – factors associated with chronic pain and severe pain

	Variables associated with Chronic pain		Variables associated with moderate pain	Variables associated with severe pain
	Univariate [OR (95% CI)]	Multivariate [OR (95% CI)]	Multivariate [OR (95% CI)]	Multivariate [OR (95% CI)]
BMI (Low-normal BMI reference category)	1.0	1.0	1.0	1.0
Overweight	1.14 (1.02–1.27)	1.08 (0.95–1.22)	1.30 (1.04–1.63)^*^	1.09 (0.87–1.34)
Obesity	1.97 (1.69–2.30)	1.59 (1.33–1.91)^**^	1.39 (1.02–1.89)^*^	1.53 (1.17–2.01)^**^
Age (65–74 y, reference category)	1.0	–	1.0	1.0
75–84 y	1.04 (0.93–1.16)	–	1.11 (0.89–1.40)	1.31 (1.05–1.63)^*^
85+ y	1.05 (0.90–1.22)	–	1.81 (1.26–2.60)^**^	1.66 (1.22–2.25)^**^
Gender (1 = female)	1.66 (1.50–1.84)^**^	1.40 (1.24–1.58)^**^	1.33 (1.06–1.67)^*^	–
Marital status (1 = married)	0.91 (0.83–1.01)	–	–	–
Education (College/university, reference category)	1.0	–	1.0	–
9-year Compulsory school	1.32 (1.16–1.50)^**^	–	1.47 (1.11–1.94)^*^	–
Upper secondary school	1.13 (0.98–1.32)	–	1.33 (0.99–1.79)	–
Income per year (< 150,000 SEK, reference category)	1.0	–	1.0	–
150,001~ 220,000	0.83 (0.73–0.94)^*^	–	0.78 (0.58–1.02)	–
> 220,000	0.64 (0.57–0.73)^**^	–	0.70 (0.51–0.95)^*^	–
Smoking (never smoker, reference category)	1.0	1.0	–	1.0
Ex-smoker, *n* %	1.14 (1.02–1.27)^*^	1.21 (1.07–1.38)^*^	–	1.30 (1.05–1.60)^*^
Current smoker, *n* %	1.13 (0.93–1.36)	1.29 (1.04–1.59)^*^	–	1.33 (0.93–1.91)
High alcohol consumption (1 = yes)	1.17 (0.92–1.48)	–	–	–
History of trauma or injury accident	3.2 (2.60–3.94)^**^	2.52 (1.99–3.19)^**^	–	–
Rheumatic diseases (1 = yes)	5.92 (5.22–6.72)^**^	5.21 (4.54–5.97)^**^	–	1.34 (1.10–1.64)^*^
Cardiovascular diseases (1 = yes)	1.35 (1.22–1.49)^**^	–	1.36 (1.10–1.67)^**^	1.33 (1.10–1.64)^*^
Respiratory diseases (1 = yes)	1.82 (1.56–2.12)^**^	1.34 (1.18–1.52)^**^	–	–
Gastrointestinal diseases (1 = yes)	2.61 (2.35–3.16)^**^	1.93 (1.64–2.27)^**^	–	–
Neurological diseases (1 = yes)	1.71 (1.54–1.91)^**^	1.34 (1.18–1.52)^**^	–	–
Urogenital diseases (1 = yes)	1.53 (1.36–1.97)^**^	–	–	–
Metabolic diseases (1 = yes)	1.59 (1.43–1.91)^**^	1.27 (1.08–1.50)^*^	–	–
Cancer diagnosis (1 = yes)	1.33 (1.07–1.57)^*^	–	–	–
Depression and/or anxiety (1 = yes)	1.99 (1.61–2.45)^**^	1.46 (1.14–1.86)^*^	–	1.83 (1.32–2.53)^**^
Number of pain sites (NPS)			1.11 (1.06–1.16)^**^	1.1 (1.06–1.13)^*^
*Model Nagelkerke R* ^*2*^		*0.236*	*0.086*	*0.08*
*Model Hosmer and Lemeshow test*		*x*^*2*^ *= 7.63, P = 0.47*	*x*^*2*^ *= 10.40, P = 0.238*	*x*^*2*^ *= 6.30, P = 0.613*

Note: ^*^P < 0.05, ^**^P < 0.01

Within the chronic pain group, obesity and overweight were weakly but significantly associated with moderate pain (OR1.30–1.39, *p* < 0.05, Table 2). In comparison, obesity had stronger impact on severe pain (OR 1.53, 95% CI 1.16–2.01, *p* < 0.01). However, aged 85 and over (OR 1.66, 95% CI 1.22–2.25) and depression and/or anxiety diseases (OR 1.83, 95% CI 1.32–2.53) were more strongly linked to severe pain than being obese. Sociodemographic factors such as age, gender, education, and income levels were also significantly related to having moderate pain. Being an ex-smoker, certain co-morbidities (e.g., rheumatic diseases and cardiovascular diseases), and number of pain sites were also significant regressors of severe pain.

## Discussion

Among the older adults in our study cohort, chronic pain was more common in people categorized as obese than in people categorized as overweight or low-normal weight. A distinct difference in anatomical pain distributions to some extent reflected the negative consequence of excess weight. The well-known disparity of pain distributions with respect to sex [13, 48–51] was also found in the cohort, but this was not statistically significant across age stratifications. Our findings contribute to the growing body of evidence that being obese, but not being overweight, is closely related to chronic pain, including severe chronic pain in the older adults. Furthermore, in our aged sample, having multiple comorbidities was more strongly related to pain-related factors than excess weight.

In the context of the current obesity epidemic, the adverse health consequences of excess weight place older adults at risk for comorbidity, poor physical function, and disability. In later life, chronic pain may show a mediating effect between obesity and these consequences [52]. The literature identifies three dominant aspects that support the relationship between obesity and chronic pain. First, increased mechanical load could explain the connection between excess weight and specific anatomical pain distributions [10, 21]. In our study, this hypothesis was reasonably confirmed by a higher proportion of reported chronic pain in extremities and low back in obese olds than in normal-weight or overweight olds. Second, hyperalgesia can be the result of the gradual development of systematic chronic low-grade inflammation. For example, pain can be modulated by inflammatory processes initiated by altered levels of cytokines (C creative protein, interlukin-6, TNF-α, IL-6, adiponectin, leptin, resistin, and visfatin) in adipose tissue [10, 21, 53, 54]. Furthermore, aging contributes to a pro-inflammatory state by increasing production of cytokines and reducing the capacity to cope with a variety of stressors [55]. Our multivariate models showed that the influence of age on inflammation may contribute to the course of chronic pain and to the increase in pain intensity. Third, obese adults are more likely to suffer poor mental health [56, 57], adding to their inflammation-inducing stressors. Previous studies have recognized this co-existence and interactions between mood disorders (depression and/or anxiety) and pain [58–61]. Because the associations between these conditions are bidirectional, it is difficult to ascertain which condition causes or exacerbates the other or indeed if a causal relationship exists.

Smoking history seems to play an important role in the course of chronic pain and severe pain. As with other studies, we found that both current smoking and ex-smoking were associated with chronic pain [62–64]; however, determining the underlying mechanism is difficult as thousands of compounds in cigarette smoke produce physiological effects. Apart from the pharmacological effect of nicotine and other ligands at the nicotinic acetylcholine receptor associated with pain, smoking behaviour to some extent reflects poor mental well-being [65]. Smoking as an unhealthy behaviour is part of negative psychological profiles and it is interconnected with obesity and pain symptoms [26, 27, 65].

In the multivariate model, we found several common comorbidities (e.g., rheumatic diseases) of chronic pain that have a greater impact than obesity. In the logistic regressions, we found that other obesity-related diseases/co-morbidities – e.g., obstructive sleep apnoea (categorized as a respiratory disease), gallbladder disorder (categorized as a gastrointestinal disease), stroke (categorized as a neurological disease), diabetes (categorized as a metabolic disease), and mental illness (depression or anxiety) – exhibited a modest association with chronic pain. These impacts may have been stronger if these diseases/co-morbidities had been asked for specifically and not included in broader categories as in the present study. Therefore, in an aging population with a high prevalence of multiple morbidities, we need to consider the above common comorbidities when evaluating chronic pain and suffering. In addition, these comorbidities are not the same comorbidities younger people experiece when suffering from chronic pain. For example, unlike a chronic pain study of younger people [26], two common obesity-related illnesses, cancer and cardiovascular disease, were not significantly associated with chronic pain in our study population. This difference suggests that the relationship between these conditions and chronic pain in old age are weaker or even absent. It may be that these common comorbidities are also age-related, so they may frequently occur in older adults with or without excess weight and pain suffering.

The analysis of these factors together with weight status provides a subset of the aging population who may be more likely to have chronic pain. To meet the particular needs of pain management for older people (e.g., pharmacological treatment, lifestyle interventions, psychological support, and rehabilitation) [66], health professionals should identify the risk group. Our study results support the growing evidence of the pain-obesity relationship in older adults. The multivariate models strongly indicate the need to have a multifactorial approach to chronic pain when assessing patients with pain. Our results indicate the need to incorporate weight/BMI as one of several factors in such assessments. It is important to be aware that not all risk factors in the models are modifiable – i.e., increased age, female gender, education, income, and having trauma history. Alternatively, the modifiable factors such as obesity, smoking, and certain chronic diseases present the opportunities for intervening. Because more modifiable factors existed in patients with severe pain than in patients with moderate pain, patients with severe pain have more interventions available to them that can relieve their pain. Healthcare professionals need to pay attention to existing multifactorial characteristics so they can identify the people at greatest risk and in most need of interventions. Therefore, pain interventions for older adults should direct their focus on the modifiable risk factors.

One important limitation of this cross-sectional study was the bias from self-reported anthropometric measures. Despite a high correlation between self-reported and measured values, the underestimation of excess weight by self-reported values has been reported [36, 37]. If we consider this bias (men by 1-unit and women by 1.19-unit underestimated BMI) [37], a total of 1117 individuals could be misclassified (28.5% of normal-weight and 14.5% of overweight). When we recalculated the regression analysis using these revised numbers, the estimates in the model did not show significant changes (data not shown).

Another weakness of our study could be the lack of generalizability of the results due to the rate of non-participants in the study population, with a valid response of 62.7% (10,000 subjects selected) [34]. The non-participants may represent a group with severe illness (e.g., admitted in hospital, residing in nursing homes, and unable to answer the questionnaire due to cognitive impairment) or a more healthy group (e.g., without any pain discomforts) so they were not interested in participating in the study [67].

In addition, our cross-sectional analysis did not uncover any causation. We cannot identify a direct cause-and-effect relationship between these factors. The results should be tested longitudinally in future studies. Additionally, although many important co-variates were included in the regression analysis, some potentially meaningful co-variates such as dietary intake and physical activity were not collected in this study. Previous studies demonstrated that unhealthy excess food intake and sedentary living cause weight gain and systemic inflammation [68, 69]. To some extent, this understanding provides for the possibility that interventions could break down the vicious circle of excess weight and chronic pain conditions. A follow-up study or longitudinal studies measuring changes could validate the impacts of changes on comorbid obesity and pain.

## Conclusion

Chronic pain affects more obese older adults than their low-normal or overweight peers. Excess weight (BMI 30 or above) is a potentially modifiable factor but not the only risk factor that is associated with chronic pain and severe pain. It is important for healthcare professionals to understand the multiple factors involved in the complex relationship between pain and excess weight. Healthcare professionals and policy makers should address the management of the coexisting modifiable factors instead of focusing on a single achievement (i.e., weight reduction). Future research should investigate the effectiveness of interventions that treat comorbid pain and obesity in older adults.

## Abbreviations

BMI: Body Mass Index
CI: Confidence Interval
NPS: Number of Pain Sites
NRS: Numeric Rating Scale
OR: Odds ratio
